# Dihydromyricetin promotes longevity and activates the transcription factors FOXO and AOP in *Drosophila*

**DOI:** 10.18632/aging.202156

**Published:** 2020-12-03

**Authors:** Xiaolan Fan, Yao Zeng, Ziqiang Fan, Liang Cui, Wenhao Song, Qi Wu, Yue Gao, Deying Yang, Xueping Mao, Bo Zeng, Mingwang Zhang, Qingyong Ni, Yan Li, Tao Wang, Diyan Li, Mingyao Yang

**Affiliations:** 1 Institute of Animal Genetics and Breeding, Sichuan Agricultural University, Chengdu, Sichuan, P. R. China; 2 Farm Animal Genetic Resources Exploration and Innovation Key Laboratory of Sichuan Province, Sichuan Agricultural University, Chengdu, Sichuan, China

**Keywords:** Dihydromyricetin, AKT, anti-aging, FOXO, ERK

## Abstract

Drugs or compounds have been shown to promote longevity in various approaches. We used *Drosophila* to explore novel natural compounds can be applied to anti-aging. Here we reported that a flavonoid named Dihydromyricetin can increase stress that tolerance and lipid levels, slow down gut dysfunction and extend *Drosophila* lifespan. Dihydromyricetin can also lessen pERK and pAKT signaling, consequently activating FOXO and AOP to modulate longevity. Our results suggested that DHM could be used as an effective compound for anti-aging intervention, which could likely be applied to both mammals and humans.

## INTRODUCTION

Aging is considered to be an important risk factor for many diseases such as cancer, Type 2 diabetes and Alzheimer's disease [1]. Many strategies have been used to delay aging, such as caloric restriction [2] and the development of FDA-approved drugs including rapamycin and metformin [3, 4]. In addition, many natural compounds have drawn the attention of scientists focusing on anti-aging. For example, the naturally-occurring plant polyphenol resveratrol could extend lifespan by reducing insulin-like growth factor-1 (IGF-1) levels and increasing AMP-activated protein kinase (AMPK) [5]. A senolytic cocktail, the natural flavonol quercetin plus dasatinib, can eliminate senescent cells [6]. Senolytics had also been proved to promote the healthspan and lifespan in mice [7].

In the aging process, the insulin/IGF-1 signaling (IIS) pathway plays an important role in the regulation of longevity. Although mutants in the IIS pathway are always deficient during development, repressing the activity of this pathway can extend various lifespans from fruit fly to mouse [8]. IGF-1 is involved in the regulation of energy metabolism. The mutant animals revealed key molecules for longevity in IGF signaling, including mechanistic target of rapamycin (mTOR) and forkhead box, subgroup O (FoxO) transcription factors. Mutant of insulin receptors (daf-2 in C. elegans), akt or genes of downstream mTOR can significantly prolong the adult lifespan in worm, fruit fly and mouse [9]. FOXO protein has been thought as one of the most important transcriptional effector of the IIS signaling. Under favorable conditions, IIS is active and causes AKT and SGK kinases to phosphorylate FOXO, resulting in its retention by the 14-3-3 protein in the cytoplasm. However, under various stress conditions, such as low IIS, starvation, heat, ultraviolet, or oxidative stress, FOXO is released from the 14-3-3 protein, then enters the nucleus to regulate the expression of stress resistance and promote longevity target genes [10]. Besides its function in stress resistance and longevity, FOXO is involved in a wide range of crucial cellular processes regulating metabolism, cell cycle arrest, and apoptosis [11]. Over twenty of DAF-16/FOXO target genes have been reported to influence aging and longevity [12]. Regulation of FOXO activity has been shown to significantly promote longevity from C. elegans to mice [11]. Therefore, FOXO acts as a major contributor for longevity and health span in the organisms.

The Ras-extracellular signal regulated kinase (ERK) is a mitogen-activated protein kinase that regulates a variety of proteins primarily by phosphorylating serine/threonine-valine (S/T-P) residues. The ERK cascade is involved in physiological responses, various physiological functions and diseases [13]. ERK1/2 can act on hundreds of targets [14]. Therefore, inhibition of the activity of ERK has a profound effect on cellular senescence and aging. Treatment with drug Trametinib, a highly specific inhibitor of Ras-Erk signaling, in Drosophila adult led to the extension of fly lifespan [15]. Similarly, reducing hippocampal ERK and p38 activation has been demonstrated to improve memory of middle-aged rats [16].

Dihydromyricetin (DHM) is a natural flavonol from Chinese herbal medicine and provides a wide range of health benefits including anti-oxidant, anti-inflammatory, and anti-carcinogen effects [17]. The role of DHM has been explored in different in vitro oxidative damage and neuroinflammatory systems, and preliminary studies have been performed in several animal models of neurodegenerative diseases, such as Alzheimer's disease [18], Parkinson's disease [19], and Huntington's disease [20]. Previous studies demonstrated that DHM could ameliorate high-fat-induced skeletal muscle insulin resistance by autophagy induction through the AMPK signaling pathway [21]. It also exhibited anti-carcinogen activity and induced apoptosis in human hepatocellular carcinoma cells. Metabolomics reveal that targets of DHM management are glucose metabolism, the TCA cycle, amino acid metabolism, purine, and pyrimidine metabolism [22].

Current studies on DHM mainly focus on its effects on diseases, however the role of DHM in the context of natural aging has not been fully understood. Given the impact on cell division and aging-relevant processes, we decided to test DHM for a possible impact on aging. In this study, we used *Drosophila* as animal model to uncover the role of DHM during the aging process. Results showed that DHM can extend lifespan and healthspan, and delay muscle and intestinal senescence, in addition to improving autophagy in *Drosophila*. DHM can inhibit the pERK and pAKT signaling, consequently activating FOXO and AOP. As the genetic mechanisms of aging in *Drosophila* and mammals are similar, the results imply that DHM could be a promising candidate for anti-aging interventions in mammals.

## RESULTS

### DHM can extend the lifespan of *Drosophila*

**Figure 1:**
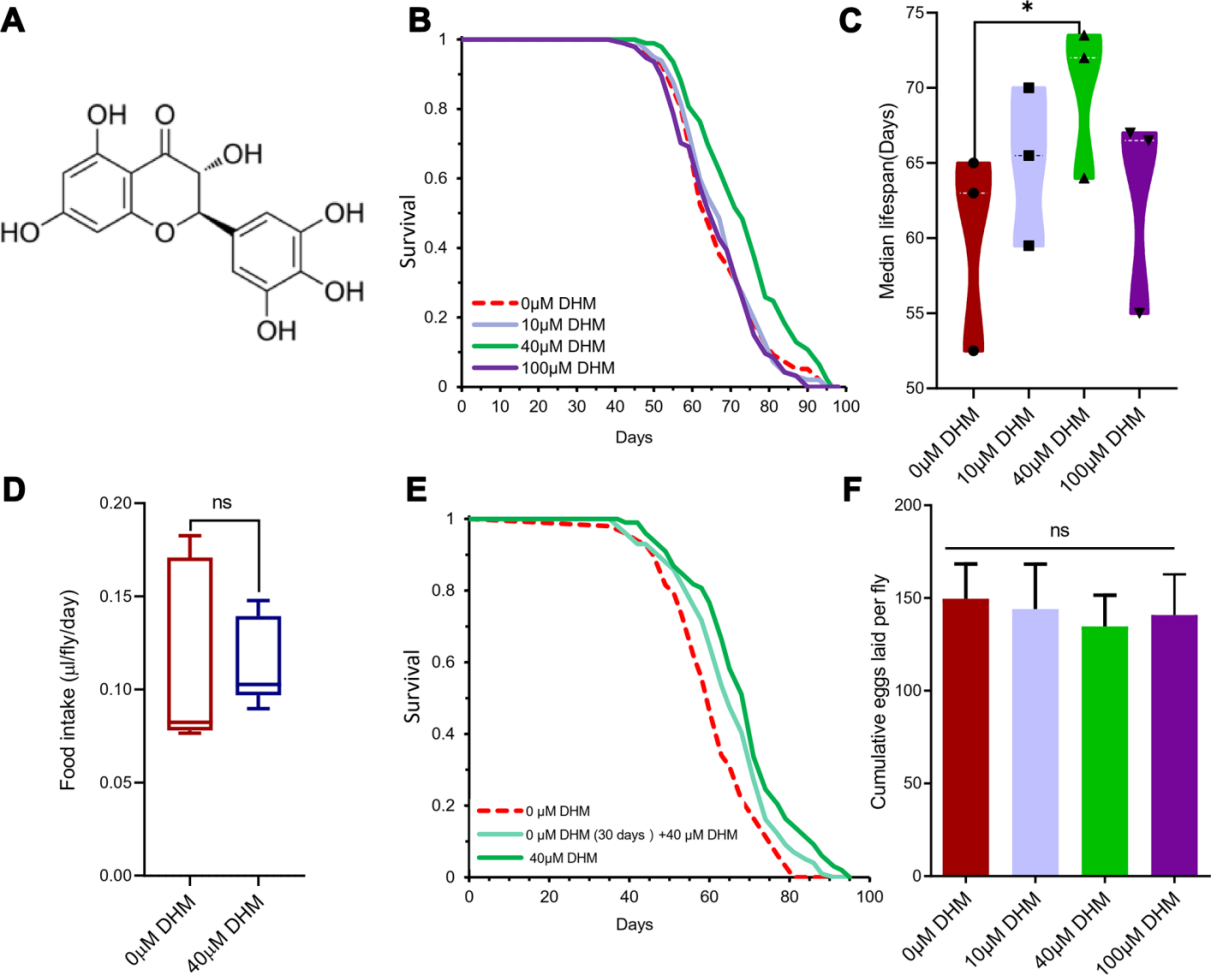
**DHM can extend the *Drosophila* lifespan.** (**A**) The structure of DHM. (**B**) DHM treatment extends the lifespan of *w^Dah^* females. Compared to flies on control food (0μM DHM), flies on 40μM of DHM food have increased median lifespans (*p*=0.00004, log-rank test - see Supplementary Table 1). (**C**) The median lifespans of the different concentrations of DHM-treated female flies. The 40μM of DHM extended the median lifespan by 16.07% (from 60.16 to 69.83 days, *p*=0.02, t-test). The average of 3 independent lifespan assays. The bars are the SE of three repeat experiments. (**D**) The food intake of the fruit fly on food without and with DHM (ns means “No significant difference”). (**E**) Lifespan curves of female flies with later-life and life-long treatment on DHM (*p*=0.0034 and *p*=1.32× 10^-6^, log-rank test - see Supplementary Table 1). (**F**) The fecundity of female flies on DHM treatment (*p*= 0.571, *p*=0.0779 and *p*=0.347; n=100; t-test).

Dihydromyricetin (2R, 3R)-3, 5, 7-trihydroxy-2- (3, 4, 5-trihydroxyphenyl)-2, 3-dihydrochromen-4-one (PubChem CID: 161557 and ChEBI: 28429) is a flavanonol, one of the subgroup of flavonoids (Figure 1A). Lifespan is an important indicator of the aging process. We firstly examined the effects of *Drosophila* lifespan by adding DHM to diet at concentrations of 0μM, 10μM, 40μM, and 100μM. The result showed that 40μM DHM can extend median lifespan by 16.07% (from 63 days to 72 days), and maximum lifespan of female flies on SYA fully fed (FF) media (Figure 1B, 1C and Supplementary Figure 1A, Supplementary Table 1A). In addition, we also tested whether DHM can extend lifespan in dietary restriction (DR) conditions. When 40μM of DHM was added into DR and FF yeast food media, it did extend the lifespans on both diets (Supplementary Figure 1B). In the following experiments, it is assumed that we chose 40 μM on FF media as the optimal working concentration for the subsequent experiments unless otherwise stated.

To measure whether the lifespan benefits from DHM were indirectly caused by the drug being aversive and/or repellant and thus causing flies to self-impose DR, we assessed the effect of the drug on food ingestion. Used by the dye-labeled food and excreta quantification (EX-Q) method for measuring fly’s food intake, there was no significant difference in food intake between DHM-treated and control group (Figure 1D). During the one hour feeding assay, DHM had no effect on food consumption (Supplementary Figure 1C). This data demonstrated that the lifespan extension by DHM was not dependent on DR.

It is not practical to take the drug during a human’s entire lifetime, therefore we tested the drug effects of only late-life administration on *Drosophila*. One group was fed FF medium as a control, and another group was fed FF food without DHM for the first 30 days of adult life, after which they were transferred to and maintained on medium containing 40μM of DHM. A further group of flies were fed on medium containing 40μM of DHM for life. We found that lifespan with later-life DHM treatment was significantly extended compared to the control group (Figure 1E and Supplementary Table 1B), however it was not as high as the group with life-long DHM treatment. The later-life DHM treatment increase of median lifespan was 8.474% compared to the life-long increase of 17.80% (Supplementary Table 1B).

Unlike rapamycin or DR treatment, lifespan extension was not associated with a reduction in fecundity [24]. We did not observe significant reduction of fecundity after treatment with DHM (Figure 1F). This result suggests a mode of action that is at least partially distinct from that of rapamycin and DR. Taken together, we demonstrate that DHM can extend the lifespan of *Drosophila*.

### DHM improves stress tolerance, lipid levels and climbing ability

**Figure 2:**
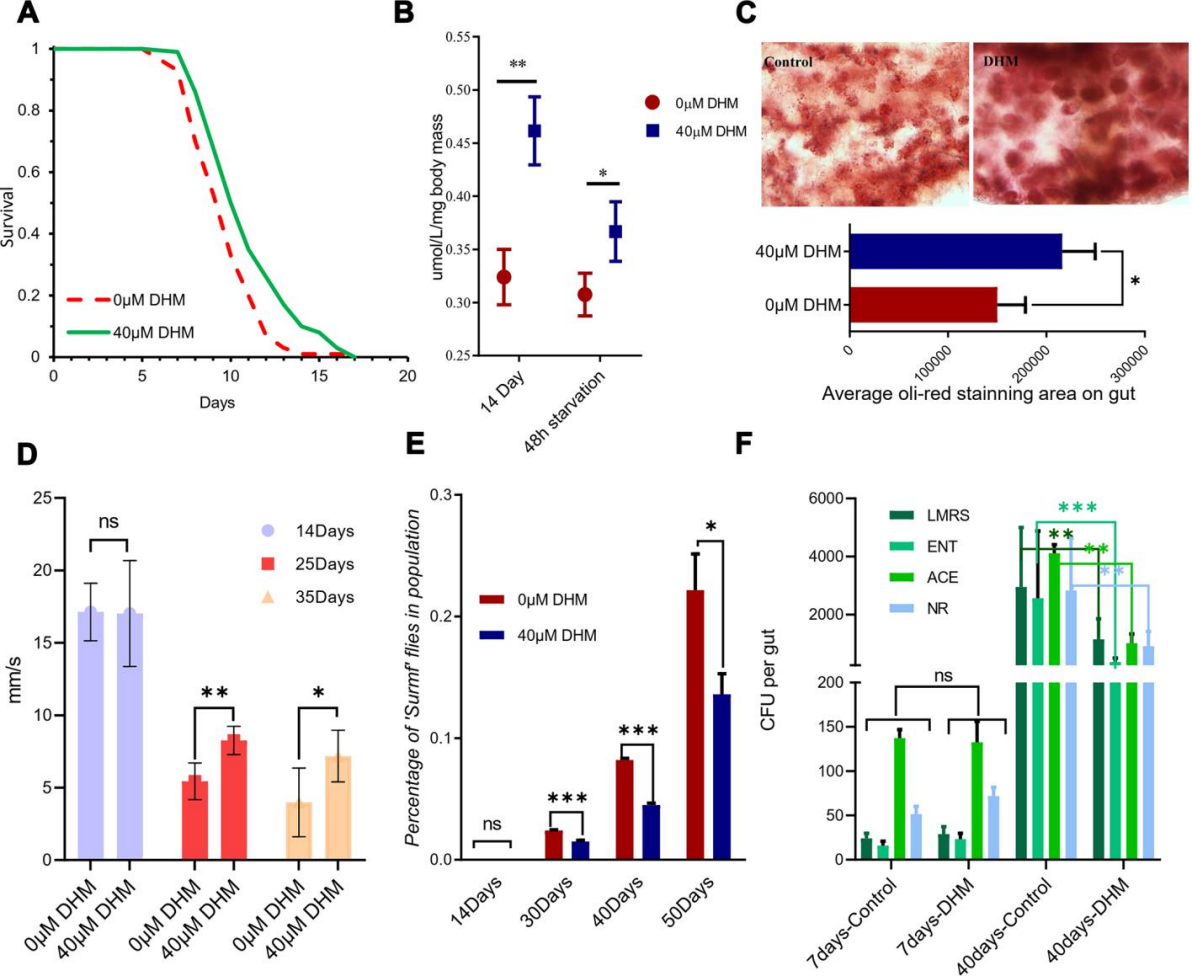
**DHM can improve stress tolerance and delay the intestinal dysfunction.** (**A**) Survival curve of fly starvation on the 1.5% agar food, showing DHM improves starvation resistance (*p*=2.7x10^-5^, log-rank test). (**B**) TAG levels of *w^Dah^* flies that were measured after 14 days of DHM treatment. (**C**) Oil red O staining of 14 days old fly guts. (**D**) *Drosophila* climbing ability from 10 days to 35 days (ns means “No significant difference”, * means *p*<0.05, **means *p*<0.01). (**E**) The percentage of ‘‘Smurf’’ flies. DHM treatment improves intestinal integrity in aged flies. (***p < 0.001, n >90 females for each genotype). (**F**) The numbers of colony-forming units (CFUs) in intestinal extracts of control and DHM-treated flies (ns means “No significant difference”, * means *p*<0.05, **means *p*<0.01; n=10; repeat=3), NR: Nutrient-rich medium, LMRS: *Lactobacilli*, ACE: *Acetobacteria*, ENT: *Enterobacteria*.

Interventions that extend lifespan are often associated with resistance to various stresses [23], we therefore tested DHM-treated flies for survival under starvation stress. Flies were treated with DHM for 10 days and then transferred to agar-only food for starvation assays. DHM treatment significantly increased survival rates under starvation compared to the control group (Figure 2A and Supplementary Table 2A).

The Triacylglyceride (TAG) level was increased in DHM-treated flies. Even after 48 hours of starvation, DHM-treated flies maintain a high TAG level (Figure 2B). We also used the Oil Red O staining to mark the stored lipid in the gut. Fly guts with DHM treatment show expansion in the oil-red staining area after 14 days (Figure 2C), demonstrating that DHM can increase the lipid store levels in *Drosophila*. DHM also significantly slowed down the climbing ability declining during aging (Figure 2D). Overall, the addition of DHM can improve the fly’s general health during its lifespan.

### DHM slows down gut dysfunction

In both *Drosophila* and mammals, gut tissue maintenance is extremely important to help maintain physical barrier integrity and appropriate immune function [24, 25]. Gut dysfunction is always associated with aging [26], therefore maintenance of gut homeostasis is very important for animals in slowing down the aging speed. We subsequently investigated the effect of DHM on gut barrier function with the “Smurf” assay [27]. The results show that gut barrier function decreases during the aging process, but that DHM treatment delays this dysfunction (Figure 2E).

Gut microbiota can also become harmful with aging. In aged flies, the load and variety of gut microbes increase, perhaps as a consequence of immune dysregulation. This dysbiosis impairs gut function, ultimately driving mortality [28]. Our previous reports and another study have shown that drugs [27] and DR can delay the microbiota intestinal dysfunction. For this study, we also observed significantly reduced microbiota in the DHM-treated aging guts, but no significant difference of the intestinal microbes in the young flies (Figure 2F). Taken together, all of the data indicates that DHM can maintain intestinal balance during the aging process.

### DHM upregulates the activity of FOXO and decreases pAKT

**Figure 3:**
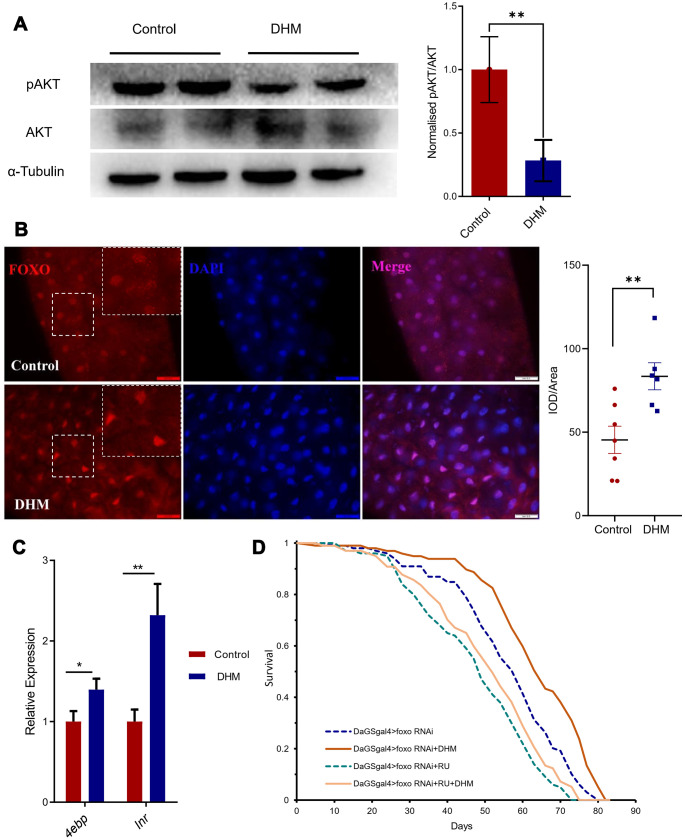
**DHM promotes FOXO activity.** (**A**) The protein expression levels of pAKT, AKT by Western blots. The bar graph illustrated the densitometry of the blots (*p*=0.031, n=2, repeat=3). (**B**) dFOXO antibody staining in the guts. The nuclei are stained with DAPI in blue, the white box shows the zoom of the corresponding position. The intensity quantifications of FOXO/Area of DAPI are shown as bar plots (n=7, 6 *p*=0.0072, t-test and the data are presented as the mean ± SEM). (**C**) The FOXO target genes *4ebp* and *inr* expression levels (relative to *actin-5C*) in the DHM-treated flies and control (*p*=0.012, *p*=0.002; n=3). (**D**) Knockdown of *foxo* in the adult fly shortens lifespan. Addition of the DHM can slightly rescue the lifespan of *foxo* knockdown flies.

Furthermore, we hope to reveal how DHM regulates healthspan and longevity. Previously, it was reported DHM can downregulate AKT signaling *in vitro* [29, 30]. AKT is the effector of IIS signaling and can suppress FOXO target expression [31]. Therefore, we checked the phosphorylation level of AKT after treatment with DHM. The result showed that pAKT level did reduce in DHM-treated flies (Figure 3A), indicating that DHM represses AKT signaling in the adult fly. As pAKT can *phosphorylate FOXO, and holding it in the cytoplasm, therefore,* we checked the FOXO protein in the nucleus of adult gut cells with antibody. We found that the DHM treatment can promote the localization of FOXO in the nucleus (Figure 3B). We also found that consistent with the two target genes of FOXO, *Inr* and *4ebp* were up expressed in the DHM-treated flies compared to the control group (Figure 3C). This demonstrated that DHM can enhance the activity of the transcription factor FOXO.

We also found that fly lifespan was shortened after knock-down of *foxo* by *Da^GS^-gal4*. Results have showed that flies of DHM treatment extend median lifespan of 10.26% (from 58.5 to 64.5 days), but when knock-down of the *foxo* the DHM-treated flies only extend median lifespan of 5.21% (from 48 to 50.5 days). That means that knockdown of *foxo* can partly block the lifespan extension effect of DHM, indicating that DHM can extend lifespan partly dependent on activation of FOXO (Figure 3D). These results also suggest that DHM can modulate pAKT-FOXO signaling activity to slow down aging.

### DHM activates AOP via ERK attenuation

**Figure 4:**
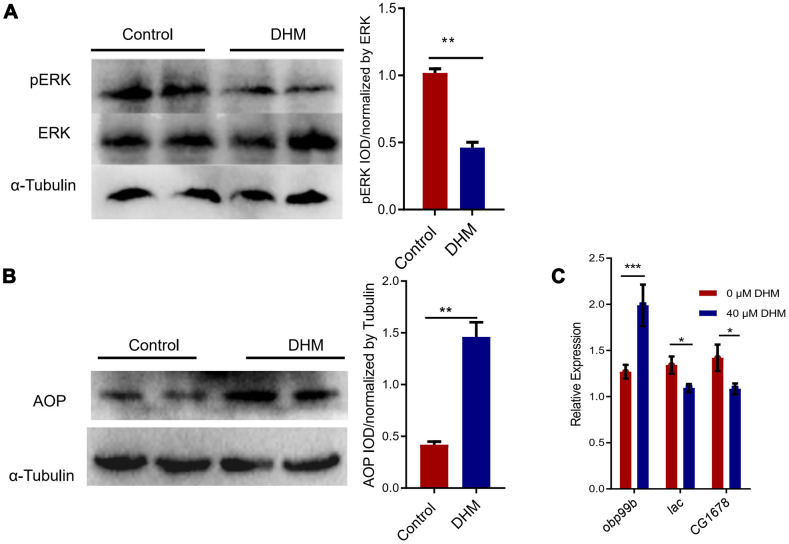
**DHM can repress pERK and activate the AOP.** (**A**) Western blots showed the pERK and ERK protein levels in the control and DHM-treated flies. The bar graph illustrated the densitometry of the blots. (**B**) Western blots showed the AOP protein levels in the control and DHM-treated adult flies. The bar graph illustrated the densitometry of the blots (*p*=0.0019, n=3). (**C**) The AOP target genes *obp99b*, *lac*, and *CG1678* expression levels (relative to *actin-5C*) in the DHM-treated flies (*p*=0.0079, *p*=0.031, *p*=0.046; n=3).

Inhibition of RAS-ERK signaling pathway was observed after DHM treatment [32]. Also inhibition of ERK signal activity was considered to be an effective anti-aging strategy [15, 33]. As we showed that knockdown of the *foxo* just partly blocked the lifespan extension effect of DHM, we then examined whether DHM affects ERK signaling in the process of regulation in aging. We confirmed that the DHM-treated flies significantly decreased the phosphorylation level of ERK by Western blots (Figure 4A). As we know that inhibition of RAS activity promotes the nuclear localization of Anterior open (AOP) in the adult fat body [15]. Therefore, we investigated if there would be AOP expression change after DHM treatment. Our results have shown that DHM led to a significant increase of AOP protein level in the adult fly (Figure 4B). To further assess the transcriptional activity of *aop* upon treatment with DHM, we also identified the activity of AOP targets. The gene *obp99b* was thought to be activated by AOP [34] and genes *CG1678* and *lac* were repressed by AOP [15]. Our qPCR results demonstrated that the expression of *obp99b* significantly increased and the expression of *CG1678* and *lac* significantly decreased in the DHM-treated flies (Figure 4C). These results indicated that DHM modulated the activity of AOP and repressed pERK to enhance health and longevity.

### DHM can improve the level of autophagy in *Drosophila*

**Figure 5:**
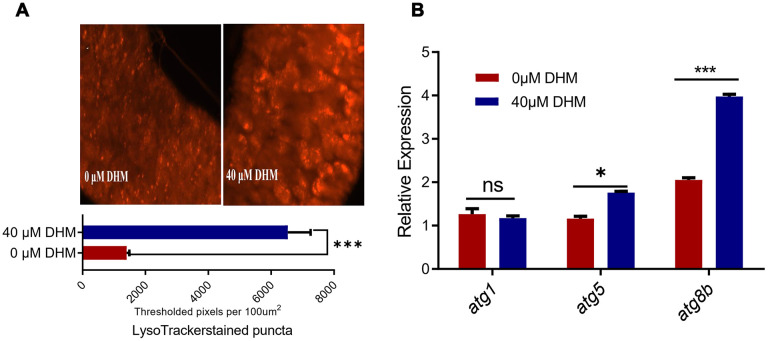
**DHM induces autophagy in *Drosophila*.** (**A**) The LysoTracker staining in the midguts of control and DHM-treated flies. The number of the lysosomes in the midguts of control and DHM-treated flies counted in per microscope field of view. (*p*=0.000023, n=6, t-test). Average number of LysoTracker-stained puncta in fly midguts isolated from control or DHM pretreated flies is presented (±SEM) (**B**) The relative expression levels of *atg1*, *agt8a* and *atg8b* in control and DHM-treated flies (*p*=0.5024, *p*=0.0013, *p*<0.000001 t-test, n=3).

Autophagy has an essential role in lifespan extension. Overexpression of *atg8a* in the neurons and muscle, or overexpression of *atg1* in neuron-specific of adult flies can extend their lifespans [35]. FOXO and AOP can regulate the expression of many autophagy-related genes [34]. Therefore, we examined the autophagy levels in the DHM-treated flies. In fly’s midgut epithelial cells, more autophagosomes were detected in DHM-treated guts compared to untreated guts by LysoTracker staining (Figure 5A). When checking the expression of genes by qPCR, we found that the expression levels of *atg8a* and *atg8b* were significantly increased in the DHM-treated flies (Figure 5B). These results demonstrated that DHM can increase the level of autophagy in vivo of *Drosophila*, which is one of the ways to effectively promote longevity.

## DISCUSSION

In this study, we uncovered that the natural compound DHM can extend lifespan and health span in *Drosophila* and repress activity of pERK and pAKT signaling in flies. We revealed the potential molecular mechanism of DHM for longevity dependent on the AKT-FOXO and ERK-AOP pathways, suggesting that DHM could be used as an effective compound for anti-aging.

Health span, which refers to the period in which organisms live without vulnerability and/or disease, is a major goal of anti-aging research [36]. Maintaining the balance and homeostasis of tissues and organs is the key to keep a health span. When DHM treatment of *Drosophila*, we noted the decline rate of the climbing ability, reflecting the level of muscle aging, was significantly lower than that in the control group (Figure 2D). The gut barrier integrity and gut microbes are closely related to intestinal homeostasis during aging [37, 38] Compared to flies without DHM-treated, the gut barrier integrity and gut microbes in the DHM treatment group decreased significantly slow (Figure 2E, 2F). Meanwhile, DHM significantly improved the starvation tolerance in flies (Figure 2A). These results indicate that the DHM-treated *Drosophila* have better health state than the control group of the same age. Therefore, it can be considered that DHM significantly prolongs the health span of fruit flies.

The FOXO is phosphorylated by AKT at three conserved residues (threonine T24, serine S256, and serine S319) and binds with 14-3-3 protein, which inhibits FOXO binding to its target DNA. This induces rapid cytoplasmic localization of FOXO proteins, resulting in inhibition of FOXO dependent transcription [39]. ERK also phosphorylates S295/345/426 of FOXO. This process showed similar functions to AKT-mediated phosphorylation, leading to enhanced cytoplasmic distribution of FOXO [40]. In this study, we found that the FOXO localizations in the nucleus were enhanced and FOXO target genes (*inr, 4ebp* and *atg5*) were up-expressed in the DHM-treated guts along with the fat body of flies. In fact, when *foxo* was silenced, we also observed a decrease in the longevity effect of DHM, indicating that the anti-aging effect of DHM at least partly depends on the activity of FOXO. Considering that RNAi could not completely silence the gene expression of *foxo*, more studies with *foxo* mutants should be performed in DHM in the future.

Inhibition of Ras-Erk signaling can extend *Drosophila* lifespan [15]. During *Drosophila* development, the key outputs of the Ras-Erk-signaling are two ETS (E-twenty six) transcription factors. A transcriptional activator named Pointed (Pnt) can be stimulated by the Ras-Erk signaling pathway and a transcriptional repressor named AOP can be inhibited by the pathway [41]. AOP and FOXO were reported to share binding locations and these two transcription factors share targets in the fat body [34]. In our case, DHM can repress the ERK phosphorylation level. At the same time, the AOP activity was upregulated by DHM treatment. Also DHM can induce autophagy in *Drosophila* in association with FOXO activity. Our results supported that ERK/AOP pathway was required for animal health and longevity.

DHM has been reported to upregulate the activity of AMPK pathways in multiple cases and can improve insulin sensitivity [42, 43]. By the AMPK pathway, it can not only regulate the aging process by integration of its signaling network, but also control autophagy through ULK1 and mTOR signaling. AMPK induces stimulation of FoxO/DAF-16, Nrf2/SKN-1 and SIRT1 signaling pathways to increase cell resistance. In addition, AMPK inhibits NF-κB signaling and inhibits inflammatory responses [44]. It is known that the important protein downstream of AMPK is the tuberous sclerosis complex (TSC), which can be also phosphorylated by multiple kinases including AKT [45] and ERK [46]. Therefore, further exploration of how DHM works on the AMPK activity could provide a better insight into DHM extending fly or human lifespan and health span.

In our experiments, we have also noticed that different fly strains led to different lifespans. This was is due to their genetic backgrounds [47]. Therefore, comparison between treatment group and control group was made for the same genetic backgrounds.

Our results demonstrate that DHM can prolong the healthspan and lifespan in *Drosophila*. It correlated to downregulating the AKT and ERK signaling and increase the activity of FOXO and AOP, eventually modifying the longevity genes, for example, genes related to autophagy and others. As we know that the signaling are conserved from *Drosophila* to human, it is suggested that DHM may also be beneficial for improved health and longevity in the human population.

## MATERIALS AND METHODS

### Fly stocks and husbandry

The wild-type stock Dahomey flies were obtained from UCLA. *Da^GS^- gal4*, *foxo-RNAi* (*CG3143* BL32427) were obtained from the Bloomington *Drosophila* Stock Center (http://flystocks.bio.indiana.edu). RNAi lines were backcrossed into *w^1118^* for at least six generations. The males of all RNAi flies were crossed with *gal4* virgin females, F1 generation females were collected and after mating, transferred to food containing 200μM RU486 (Mifepristone) for activation. All stocks were maintained at 25° C on a 12 hour: 12 hour light:dark cycle at constant humidity using SYA food [48], at 25° C on a 12 hour:12 hour light:dark cycle at constant humidity. Drugs (RU486, DHM) were added into the food after cooling it to 50° C. For all experiments, flies were reared at standard larval density and enclosed adults were collected over a 12 hour period. Flies were mated for 48 hours before sorting into single sexes.

### Antibodies and reagents

Primary antibodies used were as follows: α-Tubulin Antibody (1:2000, Sigma T9026), Rabbit anti-foxo [49] (for this study); pERK (Cell Signaling Technology, 9101); ERK(Cell Signaling Technology, 4695), AOP (Developmental Studies Hybridoma Bank, anti-yan-8b12h9); pAKT (Cell Signaling Technology, 4060); AKT (Cell Signaling Technology, 9272). Mifepristone was purchased from Sigma (M8046 Sigma). DHM (msat-120131108, HPLC≥98 %) was purchased from (Chengdu MUST Bio-Technology Co., Ltd). 30 flies were used to extract protein for Western blots analysis. Western blots were carried out with our standard protocol [27]. The corresponding bands were quantified using ImageJ software. Western blots were performed on three independent biological replicates for each genotype. The relative abundance of α-Tubulin was normalized as a loading control. The student’s *t* test was used for the statistical analysis.

### qRT-PCR

Quantitative real-time polymerase chain reaction (qRT-PCR) was performed as described before [50]. The primers listed as below:

*actin5C-5'* CTCGCCACTTGCGTTTACAGT

*actin5C-3'* TCCATATCGTCCCAGTTGGTC

*inR-5'* CATCGGAAGGGAGGCGTAA

*inR-3'* CGTTTGCCTAATCGTCGAACA

*obp99b-3'* GTTGAGGTGCACGAATCGGAC

*obp99b-5'* AGGCCTTCGTGCAGGAGTCGC

*lacosta-5'* GCTGTGGTGGTGGAAGTGCT

*lacosta-3'* GGACCTCCATCCAACCAAAC

*CG1678-5'* CGGACGCCTCACTCGGAG

*CG1678-3'* CCCAAGGTGGCAACTCATC

*4ebp-5'* ACCCTCTACTCCACCACTCC

*4ebp-3'* GGAGTTTGGCTCAATGGGGA.

### Lifespan analysis

Flies that were enclosed over a 48 hours period were collected and allowed to mate for approximately 60 hours. Females were randomly allocated to the experimental food treatments and housed in plastic vials containing food at a density of 10 flies per vial, with 10 vials per condition (n=100). Flies were transferred to a fresh food source 3 times per week, during which any deaths were recorded. DHM was dissolved in ethanol and added to SYA foods at different concentrations. Ethanol alone was added in control food. Food intake assay was carried out as our previous protocol [27]. For most lifespan assays, three independent experiments have been carried out (see Supplementary Tables 1–3).

### Smurf assay

Unless stated otherwise, flies were aged on standard*/DHM* medium until the day of “Smurf” assay [24]. Dyed medium was prepared using standard media with dyes added at a concentration of 2.5% (wt/vol) of Blue dye no.1 (Sigma-Aldrich). Flies were maintained on dyed medium for 9 hours. A fly was counted as a Smurf when dye coloration could be observed outside of the digestive tract. To calculate the Smurf Increase Rate (SIR), we plotted the average proportion of Smurfs per vial as a function of chronological age and defined the SIR as the slope of the calculated regression line.

### Immunostaining and fluorescence microscopy

*Drosophila* guts were analyzed with immunohistochemistry. The immunostaining of intestines was performed as previously described [50]. All images were captured by a Zeiss LSM780 inverted confocal microscope and processed in Adobe Photoshop and Illustrator. The staining pictures were analyzed by the Image Pro.Plus6.0. Intensity quantifications as the median fluorescence intensity of FOXO is divided by the stained median area of DAPI.

### Food intake assay

Two methods were used to measure the food intake of *Drosophila*. One is the excreta quantification (EX-Q) method developed by our lab. Flies were aged on standard/DHM medium until the 10 days, then transferred to the food intake assay medium (Erioglaucine, Sigma 861146, for dye food), and kept on the food intake vials for 24h. Then measured the food intake of flies and the data analysis as the description [51]. The other one is the “one hour feeding assay”. The control and DHM-treated flies in 10 days were used to measure the food intake as the description [52].

### Lyso-Tracker staining, imaging, and image analysis

For Lyso-Tracker staining, intact guts were removed from flies that had been maintained on 40μM DHM or control food for 20 days (n=5 flies in each group). Dissections were performed in phosphate buffered saline. Each gut was mounted into a custom-made imaging chamber and stained with 1μM of LysoTracker DS Red DND-99 (Invitrogen, Molecular Probes) for 3 minutes. Each preparation was then washed three times with PBS and mounted in mounting medium (Mounting medium, Vectashield, H1200). Imaging was performed using a fluorescence microscope with ×10 objective and ×10 eyepiece. Numbers of LysoTracker-stained puncta were analyzed by Image Pro.Plus6.0.

### Triacylglyceride assay

Flies were fed for 10 days with DHM or a control diet. Each group was divided into six repeats, every repeat containing 10 flies. Flies were then frozen in liquid nitrogen for triacylglyceride content quantification by using Triglyceride assay kit (Nanjing Jiancheng Bioengineering Institute), following the kit protocol.

### Oil Red O staining

Flies were fed for 14 days with and without DHM food. Then guts were partially dissected in PBS to expose the inner face of the epidermis and fixed in 4% paraformaldehyde/PBS for 10 min., rinsed twice with distilled water, incubated for 20 to 30 min in Oil Red O stain (6 ml of 0.1% Oil Red O in isopropanol and 4 ml distilled water: prepared fresh and passed through a 0.45-mm syringe filter). After guts were blotted by tissue paper and rinsed twice with distilled water, the stained guts were mounted in glycerol and then scored for large Oil-Red-O positive droplets.

### Measurement of vertical climbing ability

Vertical locomotion (climbing) was assessed by the rapid iterative negative geotaxis assay [27]. For each treatment group, 150 flies were placed in 10 vials. The vial was gently shaken until all flies were displaced to the bottom of the vial. Flies were then permitted to climb for 4 seconds and then photographed. Two groups (DHM-fed and controls) were assayed simultaneously. The assay was repeated five times with independent groups of flies. The distance climbed was measured in Adobe Photoshop and the speed was calculated by distances.

### Stress assays

For the starvation assay, flies were housed in vials containing 1.5% agarose to provide moisture, but no nutritional value. Deaths were recorded every day [48].

### Selective plates for bacterial cultures

10 guts homogenates from flies at 7 or 40 days of age were plated on nutrient-rich medium (NR) or on selective plates allowing growth of Lactobacilli (LMRS), Acetobacteria (ACE), or Enterobacteria (ENT). Selective plates were generated according to the previous recipes [50]. Acetobacteraceae: 25g/L d-mannitol, 5g/L yeast extract, 3g/L peptone, and 15g/L agar. Enterobacteriaceae: 10g/L tryptone, 1.5g/L yeast extract, 10g/L glucose, 5g/L sodium chloride, 12g/L agar. Lactobacilli MRS agar: 70g/L BD Difco Lactobacilli MRS Agar. Nutrient-rich broth: 23g/L BD Difco Nutrient Agar. All media were autoclaved at 121° C for 15 minutes.

### Quantification and statistical analysis

Measurements represent the mean of at least three biological replicates in all graphs and error bars represent the standard deviation. As appropriate, log-rank test, student’s *t* test, one-way ANOVA with post-hoc Dunnett’s test, were used to calculate significance. All statistical analysis was carried out using Graphpad Prism 8.00 software. For all tests, a *p*<0.05 was considered significant. Asterisks reflecting the calculated *p* values were shown above each measurement, and ns indicated that differences between measurements were not statistically significant. No data collected were excluded from any experimental or statistical analysis.

## Supplementary Material

Supplementary Figure 1

Supplementary Tables
