# Supplementary material for: Dihydromyricetin promotes longevity and activates the transcription factors FOXO and AOP in Drosophila
Source: Aging (Albany NY). 2020 Dec 3;13(1):460–76. doi: 10.18632/aging.202156 (PMC7835053; doi:10.18632/aging.202156)
Supplement: Supplementary Figure 1 [file aging-13-202156-s001.pdf]

SUPPLEMENTARY FIGURE

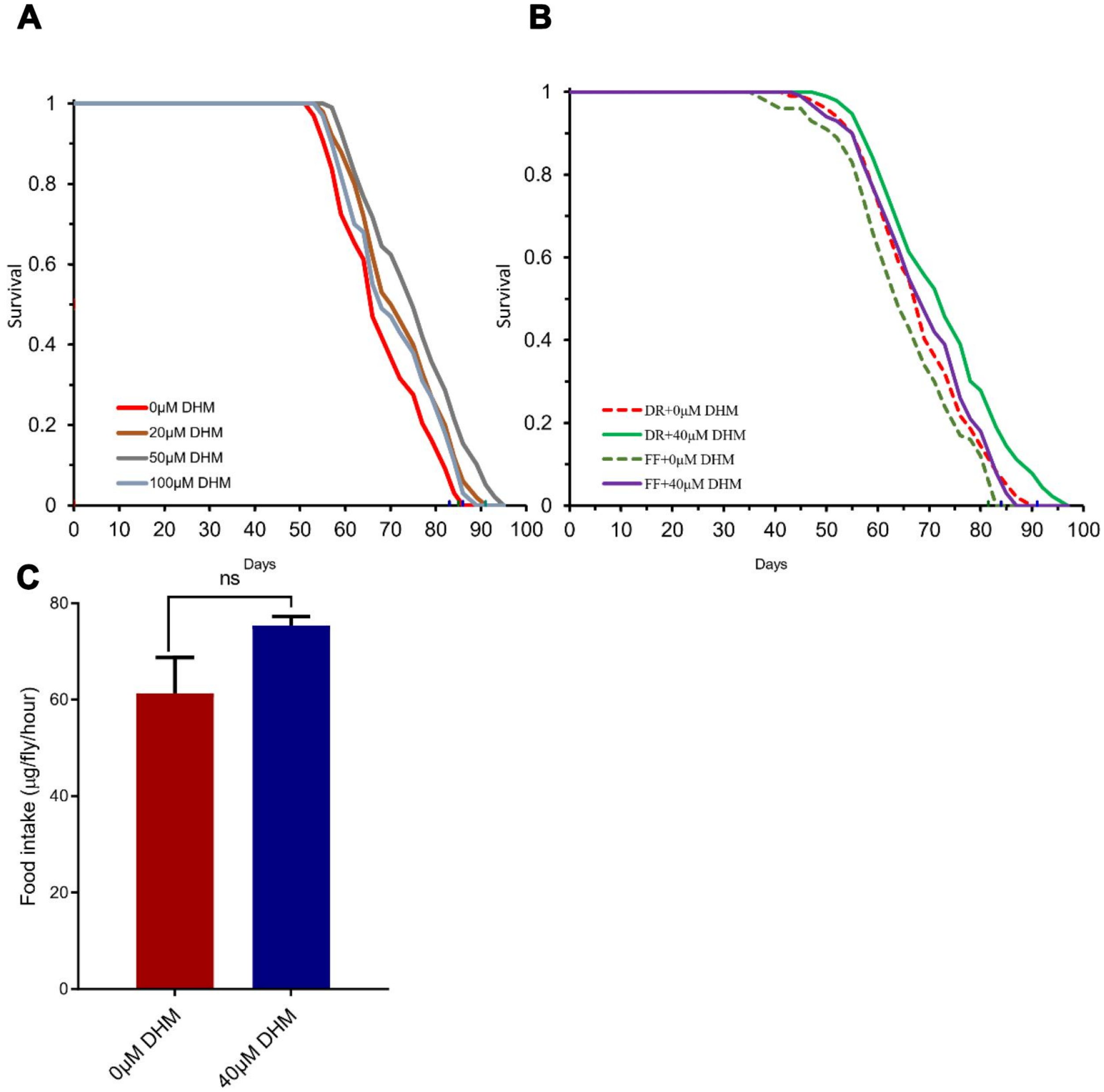

Supplementary Figure 1. DHM can extend fly lifespan. (**A**) The lifespan curves of female flies on the SYA food with 0μM, 20μM, 50μM, 100μM DHM respectively. (**B**) DHM extended fly lifespan on both fully fed (FF) and dietary restriction (DR) food (p=0.013 and p= 0.0033, log-rank test).(**C**) The food intake measured by the one hour feed assay (ns means there is no specific difference).
