# Supplementary material for: Dihydromyricetin promotes longevity and activates the transcription factors FOXO and AOP in Drosophila
Source: Aging (Albany NY). 2020 Dec 3;13(1):460–76. doi: 10.18632/aging.202156 (PMC7835053; doi:10.18632/aging.202156)
Supplement: Supplementary Tables [file aging-13-202156-s002.pdf]

SUPPLEMENTARY TABLES

Supplementary Table 1. DHM treatment extends *Drosophila* lifespans.

A, Survival data (related to Figure 1B)

Repeat 1

Genotype/ Treatment	MedLifespan (days)	MaxLifespan (days)	n Dead	n Censored	% Median Increase	p-value(log rank) vs control
**0µM DHM**	63.0	85.5	97	3		
**10µM DHM**	65.5	84.5	98	2	3.968	0.854633423
**40µM DHM**	72.0	94.0	98	2	14.289	3.76843E-05
**100µM DHM**	66.5	86.5	95	5	5.556	0.499356215

Repeat 2

Genotype/ Treatment	MedLifespan (days)	MaxLifespan (days)	n Dead	n Censored	% Median Increase	p-value(log rank) vs control
**0µM DHM**	52.5	80.5	99	1		
**10µM DHM**	59.5	83.0	100	0	13.46	0.049002524
**40µM DHM**	64.0	87.5	99	1	22.12	4.05113E-05
**100µM DHM**	55.0	83.5	100	0	4.807	0.169086376

Repeat 3

Genotype/ Treatment	MedLifespan (days)	MaxLifespan (days)	n Dead	n Censored	% Median Increase	p-value(log rank) vs control
**0µM DHM**	65.0	83.0	98	2		
**10µM DHM**	70.0	86.0	100	0	7.692	0.002907536
**40µM DHM**	73.5	91.0	100	0	13.08	9.67294E-07
**100µM DHM**	67.0	85.0	99	1	3.077	0.018283705

B, Survival data (related to Figure 1E)

Genotype/ Treatment	MedLifespan (days)	MaxLifespan (days)	n Dead	n Censored	%Median Increase	p-value (log rank) vs control
**0µM DHM**	59.0	78.0	97	3		
**0µM DHM (30 days) +40 µM DHM**	64.0	82.5	99	1	8.474	0.00345051
**40µM DHM**	69.5	89.5	98	2	17.80	1.32311E-06

Supplementary Table 2. DHM improves the tolerance of the stresses. Data are related to Figure 2.

Survival data (related to Figure 2A)

Repeat 1

Genotype/ Treatment	MedLifespan (days)	MaxLifespan (days)	n Dead	n Censored	% Median Increase	p-value(log rank) vs control
**0µM DHM**	8.5	10.5	100	0		
**40µM DHM**	9.5	13	100	0	12.5	0.013031225

Repeat 2

Genotype/ Treatment	MedLifespan (days)	MaxLifespan (days)	n Dead	n Censored	% Median Increase	p-value(log rank) vs control
**0µM DHM**	9.5	11.5	100	0		
**40µM DHM**	11	15.5	100	0	15.79	2.71896E-05

Supplementary Table 3. DHM promotes the FOXO activity. Data are related to Figure 3.

Survival data (related to Figure 3D)

Genotype/ Treatment	MedLifespan (days)	MaxLifespan (days)	n Dead	n Censored	%Median Increase	p-value(log rank) vs control
Da^{GS}gal4> foxo RNAi	58.5	76.0	99	1		
***Da^{GS}gal4> foxo RNAi*+DHM**	64.5	78.5	97	3	10.27	0.00004425
***Da^{GS}gal4> foxo RNAi* +RU**	48.0	67.0	100	0	-17.95	0.000031107
***Da^{GS}gal4> foxo RNAi* +RU +DHM**	50.5	71.5	97	3	-13.68	0.00693181
		Da^{GS}gal4>foxo RNAi +RU vs *Da^{GS}gal4>foxo RNAi* +RU+DHM			0.0936647	
